# Anticancer and Antitumor Potential of Fucoidan and Fucoxanthin, Two Main Metabolites Isolated from Brown Algae

**DOI:** 10.1155/2014/768323

**Published:** 2014-01-02

**Authors:** Soheil Zorofchian Moghadamtousi, Hamed Karimian, Ramin Khanabdali, Mahboubeh Razavi, Mohammad Firoozinia, Keivan Zandi, Habsah Abdul Kadir

**Affiliations:** ^1^Biochemistry Program, Institute of Biological Sciences, Faculty of Science, University of Malaya, 50603 Kuala Lumpur, Malaysia; ^2^Department of Pharmacy, Faculty of Medicine, University of Malaya, 50603 Kuala Lumpur, Malaysia; ^3^Department of Medical Microbiology, Tropical Infectious Disease Research and Education Center (TIDREC), Faculty of Medicine, University of Malaya, 50603 Kuala Lumpur, Malaysia

## Abstract

Seaweed is one of the largest producers of biomass in marine environment and is a rich arsenal of active metabolites and functional ingredients with valuable beneficial health effects. Being a staple part of Asian cuisine, investigations on the crude extracts of Phaeophyceae or brown algae revealed marked antitumor activity, eliciting a variety of research to determine the active ingredients involved in this potential. The sulfated polysaccharide of fucoidan and carotenoid of fucoxanthin were found to be the most important active metabolites of brown algae as potential chemotherapeutic or chemopreventive agents. This review strives to provide detailed account of all current knowledge on the anticancer and antitumor activity of fucoidan and fucoxanthin as the two major metabolites isolated from brown algae.

## 1. Introduction

Cancer is a debilitating disease which has afflicted a noticeable proportion of the entire population of the world in all generations [1]. The development of resistance to chemotherapy is considered a major hindrance to treatment of various cancers, as a notable proportion of tumors relapses and develops resistance, eventually resulting in multidrug resistance following exposure to multiple anticancer drugs with prevalent structure and mechanisms of action [2]. Furthermore, ideally anticancer agents should act exclusively against tumor cells; however, numerous chemotherapeutic drugs which are presently being used for cancer patients exhibit considerable adverse side effects on the human body, namely, bleeding, hair loss, diarrhea, and immunosuppression [3]. Hence, discovery of new natural products and metabolites isolated from microorganisms, animals, and plants possessing high efficacy against tumor cells without any toxicity on normal cells is a big leap in scientific researches. Apoptosis as a highly regulated programmed cell death has become a matter of great interest in cancer therapy and oncology because of the high potential of various chemotherapeutic agents in inducing apoptosis in a variety of cancer cells [4]. Thus, screening for natural products capable of inducing apoptosis in cancer cells that can be used alone or in combination with other chemotherapeutic drugs has now been in progress in order to elevate the therapeutic effects and reduce the side effects in cancer therapy [5].

The growing body of experimental and epidemiological evidence supporting the preventive role of marine products in controlling chronic diseases such as cancer has stimulated significant scientific interest in characterizing the active ingredients of marine products. Marine algae as part of diets and traditional remedies in the Eastern Hemisphere are still underexploited plant resources. Due to their unique living environment, algae are rich in bioactive constituents such as phycocyanin, terpenes, fucosterol, and polysaccharides [6]. Extensive beneficial health effects of marine algae have highlighted their role as a source of functional ingredients in recent years [7]. A variety of biological activities are attributed to marine algae including neuroprotection, antitumor, anticancer, antioxidant, antiobesity, anti-inflammatory, and antimicrobial, antiangiogenic, and other biological activities [7–10]. Hence, it is clearly documented that *in vivo* and *in vitro* studies with marine algae compounds continue to be extremely active in recent history [11].

Brown algae or Phaeophyceae characterized by their natural pigments form an important group of marine algae [12]. Different types of brown algae including wakame (*Undaria pinnatifida*), kombu (*Laminaria japonica*), and hijiki (*Sargassum fusiforme*) are staples in East Asians diet, especially Japan and Korea [13]. Viscous components including gepsin, porphyran, alginic acid, and oligosaccharide protecting the seaweed from invasion of bacteria that are involved in different health benefits of brown algae are clear illustrations of the importance and diversity of the constituents isolated from Phaeophyceae [14]. Thus, the active constituents of brown algae have been subjected to a variety of studies in recent years. This review briefly describes the antitumor potential of various brown algae species and summarizes the antitumor activity of fucoidan and fucoxanthin as the two main metabolites isolated from brown algae and the mechanisms underlying this activity.

## 2. Antitumor Activity of Brown Algae

The extensive research on the crude extracts of various brown algae isolated from different marine environments against different cancer cell lines shows promising anticancer potential. The study on antiproliferative activity of crude extracts of ten Phaeophyta species isolated from Brittany coasts against three human cancers, human leukaemic T cell lymphoblast (Jurkat), human Burkitt's lymphoma (Daudi), and human chronic myelogenous leukaemia (K562) cells, showed strong antitumor potential of Sargassaceae species, *Dictyota dichotoma,* and *Desmarestia ligulata* [15]. The crude methanol extract of the marine algae isolated from Aegean Shores of Turkey demonstrated strong inhibitory effect (90%) of *Padina pavonica* and *Cystoseira mediterranea* brown algae against human breast adenocarcinoma (MCF-7) and human prostate cancer cells (DU 145, PC-3, and LNCaP) [16]. The *in vitro* investigation on the enzymatic extract of *Ecklonia cava* together with its crude polyphenolic and polysaccharide fractions showed antiproliferative activity against murine colon cancer cell line (CT-26), human leukemia (THP-1), mouse melanoma (B-16), and human leukemia (U-937) cells. The polyphenolic extract showed the strongest activity against CT-26 cells (IC_50_ = 5.1 *μ*g/mL) through apoptotic cell demise according to the nuclear staining experiment [17].

The *in vivo* studies on tumor suppressive activity of brown algae signified the importance of the *in vitro* anticancer potential of these seaweeds for cancer therapy. The investigation on the antitumor activity of the powdered tissue from 21 species of air-dried brown algae against Ehrlich carcinoma in mice showed significant inhibitory effect of *S. ringgoldianum* (46.5% inhibition), *L. japonica* (57.6%), *Lessonia nigrescens* (60.0%), and *Scytosiphon lomentaria* (69.8%) after oral administration of seaweed powder (1600 mg/kg of body weight) for 28 days [18]. In another study, wakame (*U. pinnatifida*), the most popular edible seaweed in Japan, exhibited marked inhibitory effect against 7,12-dimethylbenz(*a*)-anthracene (DMBA)-induced rat mammary tumor associated with apoptosis. The results suggested induction of apoptosis via expression of transforming growth factor (TGF)-*β* through transportation of iodine from serum into mammary tissues [19]. The aqueous extract isolated from the sporophyll of *U. pinnatifida* (Mekabu) also showed strong *in vivo* and *in vitro* antitumor activity against breast cancer cells. It indicated significant antiproliferative activity against three kinds of human breast cancer cells, namely, MCF-7, T-47D, and MDA-MB-231, through an induction of apoptosis. Mekabu solution when used in daily drinking water revealed significant suppressive activity on rat mammary carcinogenesis, without any toxicity to normal cells. The inhibitory effect of Mekabu proved to be stronger than some of the widely used chemotherapeutic agents against breast cancer [20]. This remarkable anticancer and antitumor potential shown by various brown algae led scientists to isolate a variety of constituents and metabolites involved in the respective activities. The sulfated polysaccharide of fucoidan and carotenoid of fucoxanthin were found to be the most important active metabolites of brown algae as potential chemotherapeutic or chemopreventive agents.

## 3. Fucoidan

**Figure 1:**
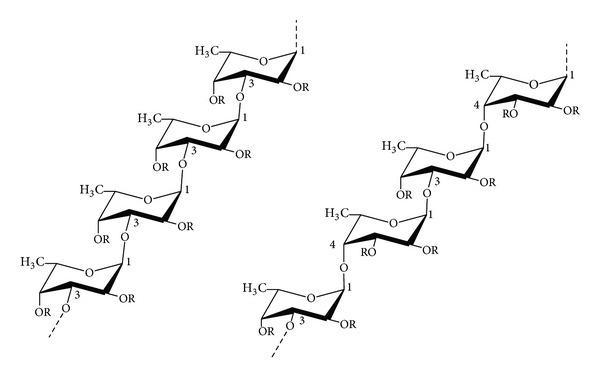
Two different types of homofucose backbone chains in fucoidans isolated from brown seaweed. **R** groups depict the potential places for attachment of noncarbohydrate (sulfate and acetyl groups) and carbohydrate (*α*-l-fucopyranose and *α*-d-glucuronic acid) substituents.

Fucoidan is a class of sulfated polysaccharides enriched with fucose in the extracellular matrix of brown algae. Fucoidans have demonstrated various biological activities including antiviral, anti-inflammatory, anticoagulant, antiangiogenic, immunomodulatory, and anti-adhesive activity [21, 22]. Due to possible differences in the chemical structure, the biological effects of this compound proved to be noteworthy depending on the species from which it is isolated (Figure 1) [22]. Thereby, fucoidan as a fucose-containing sulfated heteropolysaccharide is not uniform and its structure highly differs depending on the species source of isolation [23]. Furthermore, the constituents of fucoidan also differ with the species by small proportions of D-mannose, D-xylose, D-galactose, and uronic acid. Industrial scale of fucoidan has been prepared to be used as an additive to drinks, health foods, and cosmetics in Japan [24].

In the 1980s, the study of fucoidan in xenograft mouse models elicited antitumor and antimetastatic effects which attracted the attention of researchers in conducting more detailed studies on the antitumor and anticancer potential of this marine product [25, 26]. The antitumor activity of 31 polysaccharide fractions isolated from *S. thunbergii* when evaluated against Ehrlich carcinoma transplanted in mice revealed the antitumor potential of fucoidan or L-fucan containing roughly 30% sulfate ester groups per fucose residue, less than 2% protein, and approximately 10% uronic acid [27]. The variety of research on fucoidan revealed several critical factors involved in the marked antitumor potential of this polysaccharide.

The antiangiogenic activity of fucoidan isolated from *Fucus vesiculosus *explained, at least in part, the mechanism of the antitumor potency. Further study showed that fucoidan possessed strong inhibitory activity on tube formation following migration of human umbilical vein endothelial cells (HUVEC) and its chemical oversulfation increased the inhibitory potential. Both natural as well as oversulfated fucoidans remarkably reduced the chemotactic and mitogenic roles of the vascular endothelial growth factor 165 (VEGF_165_) on HUVEC due to antagonistic effect on the binding of VEGF_165_ to its cell surface receptor [28]. However, oversulfated fucoidan revealed stronger suppressive effect, suggesting a critical role of the number of sulfate groups in the fucoidan molecule.

Antitumor activity of fucoidan demonstrated antiproliferative activity in Lewis lung carcinoma and B16 melanoma in mice. According to their results, the number of sulfate groups in the fucoidan molecule is directly correlated with the potency of its anti-angiogenic and thereby antitumor effects [28]. In another study, enzyme-digested fucoidan extracted from Mozuku seaweed *Cladosiphon novae-caledoniae *Kylin revealed significant anti-angiogenic activity on human uterine carcinoma HeLa cells by reducing the expression and secretion of VEGF leading to attenuation of vascular tubules formation of tumor cells. Fucoidan also significantly reduced the invasion potential of human fibrosarcoma HT1080 cells possibly through suppression of the matrix metalloproteinases (MMPs) MMP-2/9 activities. It is suggested that these effects may be explained, at least partially, by the antioxidative potential of fucoidan extracts [29].

Another factor involved in the antitumor activity of fucoidan proved to be its ability to enhance the immune response. The study on fucoidan extracted from *S. thunbergii* revealed significant inhibitory effect against the growth of Ehrlich ascites carcinoma in mice (20 mg/kg per day) without any sign of toxicity. The results showed enhancement in the immune response of mice by increasing the phagocytosis activity of macrophages [30]. Prolonged survival rate of P-388 tumor-bearing mice treated with fucoidan isolated from the sporophyll of *U. pinnatifida *was associated with roughly 2-fold elevation in the IFN-gamma produced by T cells. This result suggested the involvement of IFN-gamma-activated NK cells in the antitumor activity of fucoidan [31]. In another *in vivo* study, single and repeated administration of fucoidan from *F. evanescens* (10 mg/kg body weight) indicated antimetastatic and antitumor activity in C57BL/6 mice with transplanted Lewis lung adenocarcinoma. Furthermore, treatment with fucoidan at a dose of 10 mg/kg body weight potentiated the antimetastatic effect of cyclophosphamide [32]. The chemoresistance in non-small-cell human bronchopulmonary carcinoma (NSCLC-N6) cells was significantly reduced by fucoidan extracted from *Ascophyllum nodosum *with an arrest observed in the G_1_ phase of the cell cycle. An *in vivo* study on the NSCLC-bearing nude mice also revealed the antitumor effect of fucoidan at subtoxic doses [33].

Induction of apoptosis as a critical factor in cancer therapy and oncology also contributed to significant antitumor activity of this sulfated polysaccharide. Cytotoxicity activity of fucoidan from *F. vesiculosus *demonstrated antiproliferative activity on human lymphoma HS-Sultan cell line via apoptosis induction accompanied with caspase-3 activation. The partial inhibition of apoptosis by pretreatment with pan-caspase inhibitor, Z-VAD-FMK, suggested the involvement of caspase-independent proapoptotic pathways, while the decrease in the mitochondrial membrane potential in HS-Sultan cells after 24 h treatment with fucoidan demonstrated the role of mitochondrial pathway. Furthermore, reduced phosphorylation of ERK (extracellular signal-regulated kinase) and GSK (glycogen synthase kinase) observed after 24 h treatment with fucoidan (100 *μ*g/mL) also suggested that the ERK pathways are involved in the induced apoptosis in human HS-Sultan cells [34]. Fucoidan isolated from *C. okamuranus* (containing the highest percentage of fucoidan among the brown algae) was found to reduce the damage induced by anticancer drug 5-fluorouracil (5-FU) in Hs 677.st normal stomach cells, despite minimum inhibition on the original activity of 5-FU in stomach cancer cell line (MKN45). Further study of fucoidan demonstrated antiproliferative activity on stomach cancer cells without any effects on normal stomach cells [35]. According to a more recent study, fucoidan isolated from *C. okamuranus* inhibited the proliferation of MCF-7 cells in a time- and dose-dependent manner without any effect on the viability of normal human mammary epithelial cells. Their results indicated that fucoidan induced apoptosis via caspase-8-dependent pathway which was characterized by chromatin condensation, internucleosomal DNA fragmentation, cleavage of poly(ADP ribose) polymerase, and activation of caspase-7, caspase-8, and caspase-9. Fucoidan was found to decrease cytosolic Bax and significantly elevate cytosolic cytochrome c [36]. The critical role of caspase-8 in fucoidan-induced apoptosis was indicated via inhibition of caspase-8 by the specific caspase-8 inhibitor z-ITED-FMK which resulted in the inactivation of caspase-7, caspase-8, and caspase-9 and a series of changes in cytochrome c, Bid, and Bax expression and thereby suppression in the cytotoxicity of fucoidan [36]. Since digestive enzymes in human small intestine are not able to hydrolyze fucoidan and its consumption can lead to an elevation in the concentration of luminal fucoidan within the large intestine, it may be a potent chemopreventive agent for colon cancer [37]. HT-29 and HCT116 (human colon cancer cells) treated with fucoidan (0–20 *μ*g/mL) extracted from *F. vesiculosus* demonstrated a dose-dependent antiproliferative effect and induction of apoptosis mediated through both the mitochondria-mediated and death receptor-mediated pathways. In addition, their results indicated that fucoidan induced apoptosis via the death receptor-mediated pathway through both the direct and indirect activation of caspase-3 in HT-29 cells. These results indicated that fucoidan is a potentially useful therapeutic agent for colon cancer by simultaneous activation of different apoptotic factors and pathways [38]. In human leukemia cell line (U937), the antiproliferative activity by oversulfated fucoidan was more potent compared to the native fucoidan isolated from *C. okamuranus* that greatly highlighted the role of sulfate groups in the fucoidan molecule. The results indicated that oversulfated fucoidan induced apoptosis in U937 cells through caspase-3 and caspase-7 activation-dependent pathway [24]. Furthermore, fucoidan extracted from *F. vesiculosus* induced apoptosis in HCT-15 cells (IC_50_ = 34 *μ*g/mL) via activation of pro-caspase-9 and pro-caspase-3 and downregulation of Bcl-2. Additionally, fucoidan-induced apoptosis was accompanied by the strong activation of p38 kinase and ERK and the inhibition of the phosphatidylinositol 3-kinase (PI3K)/Akt signal pathway in HCT-15 cells [39]. Fucoidan isolated from the sporophyll of Korean brown seaweed *U. pinnatifida *which is chemically defined as *O*-acetylated sulphated galactofucan also exhibited antitumor activity in A549 (alveolar carcinoma), HepG2 (hepatocellular carcinoma), HeLa (cervical carcinoma), and PC-3 cells, in a similar pattern to that of commercial fucoidan [40].

Interaction of tumor cells with platelets was found to be a critical factor in the initial steps favoring tumor metastasis [41]. Fucoidan isolated from *L. saccharina*, *L. digitata, F. serratus, F. distichus, *and* F. vesiculosus *demonstrated roughly 80% reduction in the adhesion of MDA-MB 231 tumor cells to human platelets *in vitro*. However, fucoidan extracted from *A. nodosum *and *F. evanescens* revealed 66% and 78% inhibitory effect, respectively [42]. The results proved the potential use of fucoidans for development of new drugs against tumor progression. It also proved the influence of fucoidan origin on their composition and thereby biological activities. Activation of the intrinsic and extrinsic pathways of apoptosis, increase in the immune response, suppression of angiogenesis, and reduction in the adhesion of tumor cells to human platelets are suggested as mechanisms responsible for significant antitumor activity of fucoidan (Table 1).

## 4. Fucoxanthin

Carotenoids as a group of natural pigments with more than 600 members possess a variety of biological activities including radical scavenging, immunomodulation, singlet oxygen-quenching activity, and other pharmacological effects [43]. Carotenoids include two main subclasses of nonpolar hydrocarbon carotenes and polar compounds named xanthophylls. One well known example of xanthophylls for anticancer activity is fucoxanthin [44]. Fucoxanthin (3′-acetoxy-5,6-epoxy-3,5′-dihydroxy-6′7′-didehydro-5,6,7,8,5′,6′-hexahydro-*ββ*-caroten-8-on) with a unique carotenoid structure including an allenic band and a 5,6-monoepoxide is a major nonprovitamin A carotenoid isolated from brown seaweed [13]. This orange-colored pigment carotenoid contributes more than 10% of the total carotenoids in nature, particularly in the marine ecosystem, albeit, besides brown algae, fucoxanthin was also isolated from diatoms (Bacillariophyta) [45].

Free radical scavenging activity of fucoxanthin was suggested to be the mechanism underlying its anticancer effect. An *in vitro* study by Okuzumi and colleagues [46] on the inhibitory activity of fucoxanthin against GOTO (human neuroblastoma) cell line showed 68% inhibitory effect at 10 *μ*g/mL concentration after 3 days. A decrease in the expression of N-myc gene after 4 h treatment of fucoxanthin (10 *μ*g/mL) and cell cycle arrest in the G_0_-G_1_ phase were associated with the growth reduction in GOTO cells [46]. In another *in vivo* study on duodenal carcinogenesis induced by N-ethyl-N′-nitro-N-nitrosoguanidine in mice revealed that consumption of drinking water treated with 0.005% fucoxanthin in dimethyl sulfoxide (DMSO) for 12 weeks significantly reduced the average number of tumors per mice and the percentage of tumor-bearing mice compared to the control group [47]. Further study demonstrated the inhibitory effect of fucoxanthin on liver tumorigenesis in C3H/He male mice and two-stage skin carcinogenesis in ICR mice [48]. Chemopreventive activity of fucoxanthin isolated from *Hijikia fusiforme *on the development of putative preneoplastic aberrant crypt foci (ACF) in the colon of B6C3F_1_ male mice induced by 1,2-dimethylhydrazine dihydrochloride proved the potential of fucoxanthin as a chemopreventive agent against colon carcinogenesis. Treatment with fucoxanthin (0.01%) in the drinking water of the mice for 7 weeks significantly decreased the ACF/mouse from 63.3 for the control group to 47.1 value [49]. These promising antitumor results shown by fucoxanthin warranted the various detailed investigations for determining the exact mechanisms underlying such a strong antitumor potential.

Antitumor activity of fucoxanthin isolated from *U. pinnatifida* against human leukemic HL-60 cells showed significant inhibitory effect on HL-60 proliferation in a dose-dependent manner. Treatment of HL-60 with 11.3 *μ*M and 45.2 *μ*M of fucoxanthin for 24 h reduced the viability to 46.0% and 17.3% compared to the control value, respectively. Induction of DNA fragmentation by fucoxanthin implied apoptosis for reduced proliferation of HL-60 cells [50]. The cytotoxicity of fucoxanthin isolated from *U. pinnatifida* was investigated in three lines of human prostate cancer cells, namely, PC-3, DU 145, and LNCaP. According to their results, 72 h treatment with fucoxanthin (20 *μ*mol/L) significantly reduced cell viability to 9.8% for LNCaP, 5.0% for DU 145, and 14.9% for PC-3. Induction of DNA fragmentation by fucoxanthin detected by TUNEL assay suggested that apoptosis is responsible for the reduction in cell viability of human prostate cancer cells [51]. Fucoxanthin was found to induce apoptosis in PC-3 cells via caspase-3 activation associated with reduction in the expression of Bax and Bcl-2 proteins without any change in the protein level of Bcl-X_L_. This unique response in the expression of Bcl-2 family proteins in PC-3 cells suggested a mechanism different from the other apoptosis-inducing agents which modulate the ratios of Bcl-2 and Bcl-X_L_ protein levels with the expression of Bax protein. After 48 h treatment with fucoxanthin (20 *μ*M), the level of apoptotic cells characterized by DNA fragmentation showed an increased percentage of hypodiploid cells, morphological changes, and cleavages of caspase-3 and PARP (poly(ADP-ribose) polymerase) escalated to 30% [52]. In another study, fucoxanthin isolated from *U. pinnatifida* revealed a remarkable inhibitory activity against the viability of human colon cancer cell lines (HT-29, DLD-1, and Caco-2) through apoptosis which was evidenced by DNA fragmentation. Treatment with 22.6 *μ*M of fucoxanthin in Caco-2 cells increased DNA fragmentation effect by 10-fold compared to the control which was partially inhibited by caspase inhibitor Z-VAD-fmk. Fucoxanthin also suppressed the level of Bcl-2 protein revealed by western blot analysis. Effective decrease in Caco-2 cell viability caused by a combined treatment of 10 *μ*M of troglitazone as a specific ligand for peroxisome proliferator-activated receptor (PPAR) *γ* and 3.8 *μ*M of fucoxanthin demonstrated potential complementary chemopreventive or chemotherapeutic activity of fucoxanthin on colon cancer [53].

**Figure 2:**
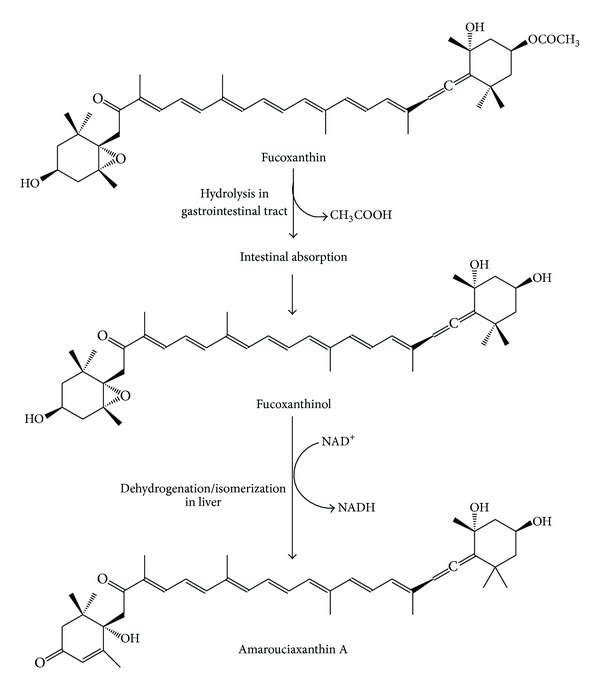
Proposed metabolic pathway of dietary fucoxanthin in mammals.

The metabolic fate of fucoxanthin in mice and HepG2 cells revealed its conversion into two metabolites, fucoxanthinol and amarouciaxanthin A. It is suggested that prior to absorption in the intestine, fucoxanthin is hydrolyzed into fucoxanthinol in the gastrointestinal tract which is later converted to amarouciaxanthin A in the liver. The proposed metabolic pathway of dietary fucoxanthin in mammals is presented in Figure 2. In another study, fucoxanthin, fucoxanthinol, and amarouciaxanthin A demonstrated IC_50_ values of 3.0 *μ*M, 2.0 *μ*M, and 4.6 *μ*M, respectively, suggesting that fucoxanthinol was the most active metabolite of fucoxanthin against PC-3 cells [13]. Further *in vitro* study of fucoxanthin isolated from *U. pinnatifida* (5 *μ*M) showed significant inhibitory effect against HCT116 cells, although it did not significantly reduce the growth of HUC-Fm (human male umbilical cord fibroblast) and MRC-5 (human normal embryonic lung fibroblast) as normal cells. The results suggested the safety of fucoxanthin against normal cells at 5 *μ*M concentration which is cytotoxic against cancer cell lines. Nevertheless, at the concentration of 10 *μ*M, fucoxanthin exhibited a significant dose-dependent growth reduction in HCT-116 cells as well as normal cells, HUC-Fm, and MRC-5 [54]. It also showed significant inhibitory effect on the proliferation of WiDr cells after 24 h in a dose-dependent manner.

Mechanistically, fucoxanthin against HCT-116 and WiDr cells demonstrated cell cycle arrest at G_0_/G_1_ phase via upregulation of p21^WAF1/Cip1^ (cyclin-dependent kinase inhibitor protein) as a mechanism for the antiproliferative and apoptosis inducing potential of fucoxanthin which may be related to the antitumorigenic effect [44]. Treatment with fucoxanthin (25 *μ*M) after 24 h also reduced the phosphorylation of the retinoblastoma protein (Rb) at Serine 807/811 and Serine 780 in WiDr cells which is a crucial factor for the progression of G_1_ phase and the transition of G_1_ to S phase. Nonetheless, at a concentration of 25 *μ*M, no change was reported in the protein levels of the cyclin-dependent kinase (cdk) 4 and D-types of cyclin, whose complexes are attributed to the phosphorylation of Rb at these sites, while treatment at concentrations higher than 50 *μ*M of fucoxanthin reduced their protein expression. These molecular changes led to apoptosis in colon cancer cells at concentrations higher than 50 *μ*M after 48 h treatment [44]. Investigation of the precise mechanism for the inhibitory activity of fucoxanthin against prostate cancer DU145 and human hepatocellular carcinoma HepG_2_ cell lines when assessed by DNA microarray and flow cytometry revealed induction of G_1_ arrest and GADD45A gene expression, a cell cycle related gene, without induced-apoptosis. Additionally, fucoxanthin also induced GADD153 and PIM1 (coding a Ser/Thr kinase) expressions in HepG2 and DU145 cells, although CYP1A1, an activator of pro-carcinogens, was significantly expressed in HepG2 cells [55]. In addition, the study on the molecular mechanism of the inhibitory effect of fucoxanthin (25 *μ*M) against HepG2 cells revealed induction of cell cycle arrest at G_0_-G_1_ phase. This inhibitory effect was associated with suppression in the phosphorylation of Rb at Serine780 and downregulation in the kinase activity of cyclin D and cdk4 complex, which is responsible for the phosphorylation of Rb at Ser780 site. Cyclin D suppression was attributed to both the transcriptional repression and protein degradation assessed by RT-PCR and western blotting analysis [56].

Human T-cell leukemia virus type 1 (HTLV-1) infection is responsible for the Adult T-cell leukemia (ATL) which is a fatal malignancy of T lymphocytes [57]. Fucoxanthin isolated from *U. pinnatifida* and its metabolite, fucoxanthinol, showed inhibitory effect against cell viability of ATL cells as well as HTLV-1-infected T cells without cytotoxicity effect on normal peripheral blood mononuclear cells and uninfected cell lines. However, fucoxanthinol was found to be roughly two times more potent than fucoxanthin. The investigation of the mechanism responsible for this activity showed that decreasing the expression of cdk4, cdk6, cyclin D1, and cyclin D2 in association with upregulation of GADD45*α* caused cell cycle arrest at G_1_ phase [45]. Additionally, activation of caspase-3, caspase-8, and caspase-9 proved the induction of apoptosis. Nuclear factor-*κ*B and activator protein-1 were also inactivated due to suppression of I*κ*B*α* phosphorylation and JunD expression. The results were confirmed by *in vivo* study of fucoxanthinol which caused inhibition of tumor formation in mice with severe combined immunodeficiency harboring tumors induced by inoculation of HTLV-1-infected T cells [45]. The antitumor activity by fucoxanthin (50 *μ*M and 75 *μ*M) and possible mechanisms underlying the inhibitory effect in MGC-803 cells (human gastric adenocarcinoma) revealed dose-dependent elevation in the ratio of cells in G_2_/M phase and apoptotic cells. This inhibitory effect was associated with significant reduction in the expression of survivin, STAT3 and CyclinB1. The decrease in the expression of CyclinB1 and STAT3 was attenuated by combined treatment of fucoxanthin and AG490 as the inhibitor for JAK/STAT (Janus Kinase/Signal Transducer and Activator of Transcription) signal pathway while the survivin expression was increased. The results indicated the possible role of JAK/STAT signal pathway in the induction of fucoxanthin antitumor activity [58].

Besides direct anticancer activity, the *in vitro* and *ex vivo* investigations of fucoxanthin also revealed the anti-angiogenic effect of this carotenoid as a complementary potential for cancer prevention. Treatment with fucoxanthin at concentrations greater than 10 *μ*M showed significant suppressive effect on the proliferation of human umbilical vein endothelial cells (HUVEC) and tube formation without any significant activity on HUVEC chemotaxis. In addition, 10–20 *μ*M concentration of fucoxanthin markedly suppressed the formation and development of blood vessel-like structures from CD31-positive cells using embryonic stem cell-derived embryoid bodies [59]. These results suggest that fucoxanthin can be a potential suppressor of differentiation of endothelial progenitor cells into endothelial cells through disruption in new blood vessel formation. The *ex vivo* angiogenesis study of fucoxanthin and fucoxanthinol using a rat aortic ring demonstrated suppression of microvessel outgrowth in a dose-dependent manner. The noteworthy angiogenic activity of fucoxanthin might be beneficial for suppression of the cancer aggravation [59]. It is suggested that extensive antitumor activity of fucoxanthin and its metabolites which is summarized in Table 2 is possibly attributed to the presence of 5,6 monoepoxide in the fucoxanthin molecule as one of the contributing factors [60].

## 5. Conclusions

The extensive biological activities mentioned for brown algae include the transcendent anticancer and antitumor effect of fucoidan and fucoxanthin as the two crucial metabolites isolated from various species of Phaeophyceae. Activation of the intrinsic and extrinsic pathway of apoptosis, increase in the immune response, suppression of angiogenesis, and reduction in the adhesion of tumor cells to human platelets are suggested as mechanisms responsible for significant antitumor activity of fucoidan. The superiority of antitumor strength shown by oversulfated fucoidan over natural fucoidan exhibited the critical role of the number of sulfate groups in this molecule. Carotenoid of fucoxanthin and to more extent its metabolite, fucoxanthinol, demonstrated noteworthy antitumor activity associated with the free radical scavenging potential, induction of apoptosis, and the anti-angiogenic effect. It is hoped that this review would be a fountain of motivation and guidance for the interested researchers in conducting further preclinical and clinical studies leading to the development of new chemotherapeutic or chemopreventive agents derived from the metabolites of brown algae.

## Figures and Tables

**Table 1:** Anticancer and antitumor activity of fucoidans isolated from brown algae.

Source of isolation	Type of activity and possible mechanisms	References
*S. thunbergii *	Growth inhibitory activity in Ehrlich ascites carcinoma in mice	[30]
*S. thunbergii *	Antitumor effect in mice with Ehrlich carcinoma transplanted	[27]
*A. nodosum *	*In vivo* and *in vitro* inhibitory effect against NSCLC-N6, non-small-cell human bronchopulmonary carcinoma	[33]
*U. pinnatifida *	Antitumor effect against P-388 tumor-bearing mice	[31]
*F. vesiculosus *	Elevation of antiangiogenic and antitumor activities by oversulfation	[28]
*C. novae-caledoniae * Kylin	Anti-angiogenic activity on human uterine carcinoma HeLa cells	[29]
*F. vesiculosus *	Induction of apoptosis in human lymphoma HS-Sultan cell line associated with caspase-3 activation and downregulation of ERK pathway	[34]
*C. okamuranus *	Growth inhibitory activity on stomach cancer cell line of MKN45	[35]
*F. evanescens *	Enhancement in etoposide induced caspase-dependent cell death pathway on MT-4, human malignant lymphoid cell lines	[42]
*F. evanescens *	Antimetastatic and antitumor activity in C57Bl/6 mice with transplanted Lewis lung adenocarcinoma	[32]
*L. saccharina*, *L. digitata*, *F. vesiculosus*, *F. serratus*, *F. distichus*, *F. evanescens*, and *A. nodosum *	Blocked adhesion of MDA-MB-231 breast carcinoma cell to platelets	[22]
*C. okamuranus *	Induction of apoptosis in U937, human leukemia cells, by oversulfated form of fucoidan	[24]
*C. okamuranus *	Induction of apoptosis in MCF-7 cells, human breast cancer, via caspase-8-dependent pathway	[36]
*F. vesiculosus *	Induction of apoptosis in HCT-15, colon carcinoma cells	[39]
*F. vesiculosus *	Induction of apoptosis in HT-29 and HCT116, human colon cancer cells, via both intrinsic and extrinsic pathways	[38]
*U. pinnatifida *	Antitumor activity against PC-3, HepG2, A549, and HeLa cancer cells	[40]

**Table 2:** Anticancer and antitumor activity of fucoxanthin and its metabolites isolated from brown algae.

Type	Source	Type of activity	References
Fucoxanthin	*U. pinnatifida *	Inhibitory effect against growth of GOTO cells, a human neuroblastoma cell line	[46]
Fucoxanthin	*U. pinnatifida *	Inhibitory effect against duodenal carcinogenesis induced by N-ethyl-N′-nitro-N-nitrosoguanidine in mice	[47]
Fucoxanthin	*U. pinnatifida *	Suppression of skin and liver carcinogenesis *in vivo *	[48]
Fucoxanthin	*H. fusiforme*	Reduced development of putative preneoplastic aberrant crypt foci (ACF) in colon of mice	[49]
Fucoxanthin	*U. pinnatifida *	Inhibitory effect against the proliferation of HL-60, human leukemia cell line, through apoptosis	[50]
Fucoxanthin	*U. pinnatifida *	Decreased cell viability in prostate cancer cell lines of PC-3, DU 145, and LNCaP	[51]
Fucoxanthin	*U. pinnatifida *	Inhibitory effect against viability of human colon cancer cell lines, HT-29, DLD-1, and Caco-2, through apoptosis	[53]
Fucoxanthinol, amarouciaxanthin A	Metabolites of fucoxanthin	Inhibitory effect against PC-3, prostate cancer cell line	[13]
Fucoxanthin	*U. pinnatifida *	Inhibitory effect against HCT116, human colorectal adenocarcinoma	[54]
Fucoxanthin	*U. pinnatifida *	Induction of apoptosis in PC-3 cells via caspase-3 activation	[52]
Fucoxanthin	*L. japonica *	Cell cycle arrest at G_0_/G_1_ phase in human colon cancer cells induced by upregulation of p21^WAF1/Cip1^	[44]
Fucoxanthin	*Unmentioned *	Induction of G1 arrest and GADD45A gene expression in HepG2 and DU145 cells	[55]
Fucoxanthin	*U. pinnatifida *	Antiadult T-cell leukemia activity	[45]
Fucoxanthinol	Metabolites of fucoxanthin	*In vivo* and *in vitro* antiadult T-cell leukemia activity	[45]
Fucoxanthinol	*L. japonica *	Inhibitory effect against HepG2, human hepatic carcinoma, associated with downregulation of cyclin D	[56]
Fucoxanthin	*U. pinnatifida *	Induction of cell cycle arrest in G2/M phase and apoptosis in MGC-803 cells, human gastric adenocarcinoma	[58]
